# The relationship of neutrophil-to-lymphocyte ratio with health-related quality of life, depression, and disease activity in SLE: a cross-sectional study

**DOI:** 10.1007/s00296-023-05381-8

**Published:** 2023-07-05

**Authors:** Eleni Papachristodoulou, Loukas Kakoullis, Costas Christophi, Savvas Psarelis, Victor Hajiroussos, Konstantinos Parperis

**Affiliations:** 1 Department of Medicine, University of Cyprus Medical School, Nicosia, Cyprus; 2 Department of Medicine, University of Patras School of Health Sciences, Patras, Greece; 3 Department of Internal Medicine, Mount Auburn Hospital, Cambridge, MA USA; 4 Harvard Medical School, Boston, MA USA; 5 Department of Biostatistics and Epidemiology, Cyprus University of Technology, Limassol, Cyprus; 6 Department of Rheumatology, Nicosia General Hospital, University of Cyprus Medical School, Nicosia, Cyprus; 7 Ygia Polyclinic Hospital, Limassol, Cyprus; 8 Department of Internal Medicine, Division of Rheumatology, University of Cyprus Medical School, Palaios Dromos Lefkosias Lemesou No. 215/6, Aglantzia, 2029 Nicosia,, Cyprus

**Keywords:** Systemic lupus erythematosus, Depression, Quality of life, Biomarker

## Abstract

The neutrophil-to-lymphocyte ratio (NLR) emerged as a potential biomarker in SLE, but its association with several outcomes remains unclear. We aimed to evaluate the relationship between NLR and SLE disease activity, damage, depression, and health-related quality of life. A cross-sectional study was conducted, including 134 patients with SLE who visited the Division of Rheumatology between November 2019 and June 2021. Demographics and clinical data including NLR, Safety of Estrogens in Lupus Erythematosus National Assessment-Systemic Lupus disease activity index (SELENA–SLEDAI), Systemic Lupus International Collaborating Clinics/American College of Rheumatology Damage Index (SDI), physician global assessment (PhGA), patient global assessment (PGA), patient health questionnaire (PHQ)-9, patient self-rated health, and lupus quality of life (LupusQoL) scores, were collected. Patients were stratified into two groups and compared using the NLR cut-off of 2.73, the 90th percentile value of healthy individuals. The analysis included t-test for continuous variables, χ^2^-test for categorical variables, and logistic regression adjusting for age, sex, BMI, and glucocorticoid use. Among the 134 SLE patients, 47 (35%) had an NLR ≥ 2.73. The NLR ≥ 2.73 group had significantly higher rates of severe depression (PHQ ≥ 15), poor/fair self-rated health, and the presence of damage (SDI ≥ 1). These patients also scored significantly lower in LupusQoL domains (physical health, planning, and body image), and higher in SELENA-SLEDAI, PhGA, and PGA. Logistic regression confirmed that high NLR is associated with severe depression (PHQ ≥ 15) (OR:7.23, 2.03–25.74), poor/fair self-rated health (OR:2.77,1.29–5.96), high SELENA-SLEDAI score(≥ 4) (OR:2.22,1.03–4.78), high PhGA (≥ 2) (OR:3.76, 1.56–9.05), and presence of damage (SDI ≥ 1) (OR:2.67, 1.11–6.43). High NLR in SLE may indicate depression, worse quality of life, active disease, and the presence of damage.

## Introduction

The neutrophil-to-lymphocyte ratio (NLR), is an easily accessible marker, obtained by dividing the neutrophil count by the number of lymphocytes from a routine blood sample, which can indicate the balance between adaptive immunity and systemic inflammation [1]. It demonstrated a prognostic value in various conditions, including cardiovascular diseases [2], cancer [3], and infections [4]. Recent studies have shown that NLR is associated with disease activity in different rheumatic diseases, including polymyalgia rheumatica [5], ankylosing spondylitis, rheumatoid arthritis [6], and Behçet’s disease [7]. A meta-analysis of nine studies demonstrated a positive correlation between NLR and Systemic lupus erythematosus (SLE) disease activity index (SLEDAI), with a correlation coefficient of 0.429 (95%CI = 0.288–0.552, *P* < 0.001) [8]. However, there is currently no universally accepted cut-off for NLR usage in clinical practice [9–13], while its potential as a marker of other SLE outcomes, such as depression and quality of life, has not yet been explored.

Patients with SLE have a higher prevalence of depression than the general population, which significantly affects their quality of life [14]. The focus of management is primarily on achieving remission and maintaining low disease activity, while HRQoL is not adequately incorporated into current SLE therapy targets [15]. However, patients with SLE can experience diminished quality of life despite adequate treatment response [16], while HRQoL and depression are important determinants of treatment adherence and healthcare use [17, 18]. Therefore, an easily obtainable indicator of HRQoL and depression would be a valuable component of the management of SLE in daily practice.

This study aims to expand upon prior research by examining the relationship between NLR and disease activity in SLE, while also investigating its correlation with lupus-specific health-related quality of life (LupusQoL), depression, and other activity measures of SLE disease. To our knowledge, this is the first study to comprehensively evaluate the relationship between NLR and health-related quality of life or depression in SLE patients.

## Materials and methods

This cross-sectional study was performed using the STROBE reporting guidelines [19] and included patients diagnosed with SLE who presented to the Division of Rheumatology at our institution between November 2019 and June 2021. Patients were eligible for inclusion if they met the American College of Rheumatology (ACR) and/or Systemic Lupus International Collaborating Clinics (SLICC)-2012 SLE classification criteria [20] were over 18 years old and provided written informed consent. This study was conducted in accordance with the Declaration of Helsinki and was approved by the National Bioethics Committee of Cyprus.

During the clinic visit, a thorough clinical evaluation was performed, including history, physical examination, and laboratory work-up, which included blood cell counts. Patient characteristics, such as gender, and age, as well as clinical characteristics, such as body mass index (BMI) (weight (kg)/height^2^ (m^2^), and the use of glucocorticoids at the time of study inclusion, were also recorded. In addition, during this visit, each patient was administered questionnaires evaluating the quality of life, depression, disease activity, and damage.

### Physician-reported measures

#### Neutrophil-to-lymphocyte ratio

A complete blood count was performed on study inclusion to estimate the NLR and the patients were categorized into two groups based on their NLR values. High NLR was defined as values ≥ 2.73, which corresponds to the NLR values of the 90th percentile of healthy individuals [21].

#### Disease activity

Disease activity was evaluated by the physician global assessment (PhGA) for SLE activity, and the Safety of Estrogens in Lupus Erythematosus National Assessment–Systemic Lupus Erythematosus Disease Activity Index instrument score (SELENA-SLEDAI) [22].

PhGA involves the clinician’s judgment and uses a categorical scale from 0 to 3 to indicate disease activity. A score of 0 indicates no disease activity, 1 indicates mild, 2 indicates moderate, and 3 indicates severe disease activity. Scores 2 and 3 were classified as having high disease activity.

We also utilized the SELENA-SLEDAI, a validated 24-item instrument that quantifies the presence of symptoms, conditions, and laboratory findings, to evaluate disease activity in the 10 days preceding the visit [22]. Active disease was defined as a score of ≥ 4.

#### Disease damage

The Systemic Lupus International Collaborating Clinics/American College of Rheumatology (SLICC/ACR) Damage Index (SDI) was utilized to evaluate the severity of SLE-induced damage on 12 organs or systems in the last six months. These include neuropsychiatric (0–6), ocular (score 0–2), pulmonary (0–5), cardiovascular (0–6), peripheral vascular (0–5), renal (0–3), gastrointestinal (0–6), gonadal (0–1), endocrine damage (0–1), musculoskeletal (0–7), skin (0–2), and malignancy (0–2). The SDI score ranges between 0 and 46 [23] Damage was defined as a score ≥ 1.

#### Major depressive disorder (MDD) quantification

The presence and severity of depression were assessed by the patient health questionnaire (PHQ)-9, which is a validated 9-item tool that has been validated in the Greek population with SLE [24]. Patients complete the PHQ-9 to examine the presence and severity of symptoms of depressive symptoms in the last two weeks [25]. Scores range between 0–27, with higher scores indicating more severe symptoms of depression. Scores ≥ 15 indicate severe or moderately severe depression.

### Patient-reported measures

#### Patient global assessment (PGA)

Patient global assessment (PGA) was also used to assess disease activity in SLE. Patients rated their disease activity on a scale from 0 to 10, with 0 indicating no disease activity and 10 indicating maximum activity. A PGA score ≥ 7 was considered indicative of high disease activity.

#### Patient-rated health

Patient-rated health was assessed by asking patients, **“**How would you rate your current health status?”. Responses were divided into “excellent or good” versus “fair or poor” [26].

#### Health-related quality of life (HRQoL)

Health-related quality of life (HRQoL) was evaluated utilizing the LupusQol, a specific HRQoL measure for SLE that includes 34 items and 8 domains (pain, planning, physical health, intimate relationships, body image, burden to others, fatigue, and emotional health) [27]. Each domain has a score range of 0–100, with higher scores signifying better quality of life and health status.

### Statistical analysis

The data collected were retrospectively analyzed. Summary statistics with regard to demographic and clinical characteristics were presented for all patients as well as for each NLR group, using the cut-off value of 2.73. Mean (S.D.) was reported for normally distributed variables and median (q1, q3) was reported for not normally distributed variables. Comparison between groups was made utilizing the student’s *t*-test or the Wilcoxon test, as appropriate. Categorical variables were presented as frequency (%) and χ2 test of independence was utilized to compare them between the two groups. Logistic regression models were utilized, unadjusted, and after adjusting for age, gender, BMI, and the use of glucocorticoids, to further assess the associations between the high NLR group and the different outcomes considered. Odds ratios (OR) were reported with the corresponding *p*-values and 95%CIs. All tests conducted were two-tailed, and an alpha level of significance of 0.05 was used. No imputation of missing data was conducted. Statistical analyses were performed using SAS software, version 9.4.

## Results

We included a total of 134 patients with SLE enrolled in the Cyprus Lupus Registry, of whom 116 (87.2%) were females with a mean age of 48 years (Table 1). Table 2 shows the median [Q1, Q3] and the percentage of the different scores investigated in the study participants as well as in each NLR group.

**Table 1:** Demographic characteristics

	Overall		NLR < 2.73		NLR > = 2.73		*p*-value
	*N*	Mean ± S.D. or *n*(%)	*N*	Mean ± S.D. or *n*(%)	*N*	Mean ± S.D. or *n*(%)	
Age	134	48.10 ± 15.05	87	48.66 ± 14.26	47	47.09 ± 16.54	0.566
Gender (% Female)	133	116 (87.2%)	87	77 (88.5%)	46	39 (84.8%)	0.541
BMI (kg/m2)	132	24.67 ± 4.94	86	24.49 ± 4.65	46	25.02 ± 5.46	0.557
Smoking Status (% Yes)	133	35 (26.3%)	87	25 (28.7%)	46	10 (21.7%)	0.383

*S*.*D*. Standard deviation, *BMI* Body Mass Index, *NLR* Neutrophil to lymphocyte ratio

**Table 2:** Activity index-SELENA-SLEDAI, Damage index-SLICC/ACR DI, Lupus QoL, PHQ9, Patient global assessment, Self-rated health, and Physician global assessment

	*N*	Overall	NLR < 2.73		NLR ≥ 2.73		*p*-value
		Median [Q1, Q3] or *N* (%)	*N*	Median [Q1, Q3] or *n* (%)	*N*	Median [Q1, Q3] or *n* (%)	
PHQ 9	132		86		46		0.033
% Minimal depression 0–4		54 (40.9%)		35 (40.7%)		19 (41.3%)	
% Mild depression 5–9		37 (28.0%)		28 (32.6%)		9 (19.6%)	
% Moderate depression 10–14		25 (18.9%)		18 (20.9%)		7 (15.2%)	
%Moderately severe depression 15–19		9 (6.8%)		3 (3.5%)		6 (13.0%)	
% Severe depression 20–27		7 (5.3%)		2 (2.3%)		5 (10.9%)	
Depression (PHQ ≥ 10)	132	41 (31.1%)	86	23 (26.7%)	46	18 (39.1%)	0.143
Moderately Severe or Severe depression (PHQ ≥ 15)	132	16 (12.1%)	86	5 (5.8%)	46	11 (23.9%)	0.002
Lupus QoL							
Pain	131	75.0 [41.6, 91.7]	85	75.0 [50.0, 100.0]	46	66.6 [41.6, 83.3]	0.155
Planning	131	75.0 [41.6, 100.0]	85	83.3 [50.0, 100.0]	46	58.3 [33.3, 91.6]	0.010
Physical health	131	68.8 [34.3, 87.5]	85	75.0 [53.1, 93.7]	46	59.4 [28.1, 81.3]	0.012
Intimacy	60	75.0 [50.0, 75.0]	40	75.0 [50.0, 81.2]	20	50.0 [31.3, 75.0]	0.108
Body image	130	87.5 [66.6, 100.0]	85	91.6 [75.0, 100.0]	45	80.0 [55.0, 100.0]	0.028
Burden to others	130	75.0 [58.0, 100.0]	84	79.0 [58.3, 100.0]	46	75.0 [50.0, 100.0]	0.286
Fatigue	131	68.7 [50.0, 87.5]	85	62.5 [50.0, 87.5]	46	68.7 [37.5, 81.2]	0.502
Emotional health	131	79.1 [58.3, 91.6]	85	79.1 [66.6, 91.6]	46	72.9 [50.0, 88.0]	0.154
Self-rated health	134		87		47		0.001
% Poor		7 (5.2%)		2 (2.3%)		5 (10.6%)	
% Fair		43 (32.1%)		23 (26.4%)		20 (42.6%)	
% Good		67 (50.0%)		45 (51.7%)		22 (46.8%)	
% Excellent		17 (12.7%)		17 (19.5%)		0 (0.0%)	
Poor/Fair health							
% Yes		50 (37.3.%)		25 (28.7%)		25 (53.2%)	0.005
Activity index -SELENA-SLEDAI	134	2 [0, 5]	87	2 [0, 4]	47	4 [2, 8]	0.002
Active disease (SELENA-SLEADI ≥ 4)		61 (45.5%)		33 (37.9%)		28 (59.6%)	0.016
Physician global assessment	132	1 [0,2]	86	1 [0,1]	46	1 [1, 2]	< .001
PhGA (high, scores 2 or 3)		37 (28.0%)		16 (18.6%)		21 (45.7%)	< .001
Patient global assessment	134	4 [2, 5]	87	3 [1, 5]	47	5 [3.6]	0.001
PGA ≥ 7		16 (11.9%)		7 (8.0%)		9 (19.1%)	0.059
Damage index—SLICC/ACR DI	134	0 [0,1]	87	0 [0,1]	47	0 [0,2]	0.054
Damage (SLICC/ACR DI ≥ 1)		43 (32.1%)		23 (26.4%)		20 (42.6%)	0.057

*SELENA-SLEDAI* Safety of Estrogens in Lupus Erythematosus National Assessment—Systemic Lupus Erythematosus Disease Activity Index, *SLICC/ACR DI* Systemic Lupus International Collaborating Clinics/American College of Rheumatology Damage Index, *Lupus QoL* Lupus Quality Of Life, *PHQ* Patient Health Questionnaire, *PhGA* Physicial global assessment, *PGA* Patient global assessment, *NLR* Neutrophil to lymphocyte ratio

A high NLR (NLR ≥ 2.73) was detected in 47 (35%) patients, 39 (85%) of whom were females and the mean age was 47 years. There were no significant differences in demographic features between the two NLR groups (NLR ≥ 2.73 vs NLR < 2.73) (Table 1). We also compared the indices between the two groups, including PHQ-9 score, Lupus QoL domains, self-rated health, SELENA-SLEDAI, PhGA, PGA, and SDI, and the results are demonstrated in Table 2.

### NLR and depression in SLE

In regard to depression, high NLR was associated with the presence of moderately severe or severe depression (PHQ ≥ 15) (23.9% vs. 5.8%, *p* = 0.002) (Table 2). However, the differences in the frequency of depression (PHQ ≥ 10) between the two groups did not reach significance.

### NLR and general health status in SLE

Regarding the impact of SLE on HRQoL, significant differences were found between the high and low NLR groups in various LupusQoL domains (Table 2). Specifically, the high NLR group had significantly worse planning (median 83 vs 58, *p* = 0.01), physical health (median 75 vs 59, *p* = 0.01), and body image (median 92 vs 80, *p* = 0.03) domains whereas, no significant differences were identified in the domains of pain, intimacy, burden to others, fatigue, and emotional health. Additionally, a significant association was noted between high NLR and worse ratings in self-rated health, with a significantly higher portion of patients in the high NLR group rating their health status as poor or fair.

### NLR and disease activity, as well as damage in SLE

The group of patients with high NLR exhibited higher disease activity based on the various indices evaluated (Table 2). Specifically, NLR ≥ 2.73 was associated with significantly higher disease activity scores measured with the SELENA-SLEDAI, PhGA, and PGA score, Moreover, high NLR was associated with the presence of active disease as defined by SELENA-SLEDAI (≥ 4), PhGA (≥ 2), and PGA (≥ 7)) scores albeit the latter did to achieve statistical significance (*p* = 0.059). The high NLR group also displayed a trend toward a higher SDI score (*p* = 0.054) and a greater frequency of damage presence (SDI ≥ 1) (*p* = 0.057).

### The relationship of NLR with depression, general health status, disease activity, and damage adjusted for age, gender, BMI, and glucocorticoid use

The current study utilized logistic regression models to examine the relationship between NLR and various indices of disease activity (Table 3). The unadjusted model indicated a significant association between high NLR and severe depression (PHQ ≥ 15), poor or fair self-reported health, high disease activity (SLEDAI ≥ 4), and high PhGA (≥ 2). When adjusting for age, gender, BMI, and glucocorticoid use, similar results were obtained. Notably, the association of high NLR with the presence of damage (SDI ≥ 1), which was found to be nonsignificant in the unadjusted model, became significant following the adjustment. Specifically, the model adjusting for patient demographics and glucocorticoid use showed that high NLR was significantly associated with moderately severe/severe depression, defined as PHQ ≥ 15, with an odds ratio (OR) of 7.23 (95%CI: 2.03, 25.74, *p* = 0.002), poor or fair self-reported health with an OR of 2.77 (95%CI: 1.29, 5.96, *p* = 0.009), high disease activity (SLEDAI ≥ 4) with an OR of 2.22 (95%CI:1.03, 4.78, *p* = 0.043), high PhGA (≥ 2) with an OR of 3.76 (95%CI:1.56, 9.05, *p* = 0.003) and presence of damage (SDI ≥ 1) with an OR of 2.67 (95%CI:1.11, 6.43, *p* = 0.028). Additional adjustment for increased disease activity and presence of damage (SLEDAI ≥ 4 and SDI ≥ 1), demonstrated that NLR retained its association with severe depression (OR:6.99, 95%CI:1.85, 26.49, *p* = 0.004).

**Table 3:** Logistic regression analysis for the effect of NLR ≥ 2.73

	Unadjusted models			Models adjusted for age, gender, BMI, and use of glucocorticoid		
	OR	95% CI	*p*-value	OR	95% CI	*p*-value
Severe depression (PHQ ≥ 15)	5.09	(1.65, 15.74)	0.005	7.23	(2.03, 25.74)	0.002
Poor/fair self-rated health	2.82	(1.35, 5.89)	0.006	2.77	(1.29, 5.96)	0.009
High disease activity (SELENA-SLEADI ≥ 4)	2.41	(1.17, 4.98)	0.018	2.22	(1.03, 4.78)	0.043
High PhGA (≥ 2)	3.68	(1.66, 8.14)	0.001	3.76	(1.56, 9.05)	0.003
High PGA (≥ 7)	2.71	(0.94, 7.82)	0.066	2.47	(0.81, 7.53)	0.111
Damage (SLICC/ACR DI ≥ 1)	2.06	(0.97, 4.36)	0.059	2.67	(1.11, 6.43)	0.028

*OR* odds ratio, *CI* confidence interval, *PHQ* Patient Health Questionnaire, *SELENA-SLEDAI* Safety of Estrogens in Lupus Erythematosus National Assessment—Systemic Lupus Erythematosus Disease Activity Index, *SLICC/ACR DI* Systemic Lupus International Collaborating Clinics/American College of Rheumatology Damage Index, *PhGA* Physician global assessment, *PGA* Patient global assessment, *BMI* body mass index

## Discussion

The current study provides further evidence of the association between high NLR and active SLE disease, which is consistent with previous studies [8–10]. Importantly, it was also found that high NLR, using a cut-off of 2.73, which corresponds to the 90th percentile of the healthy population [21], was significantly associated with the presence of severe depression and worse health-related quality of life.

The prevalence of depression in SLE patients is higher compared to the general population [14]. Although major depression has been associated with NLR [28], this association has not been previously investigated in patients with SLE. In this study, an association was identified between the presence of severe/moderately severe depression (defined as PHQ-9 ≥ 15) and high NLR. While the disease activity has been associated with both the severity of depression [29] and NLR values [8], the association between high NLR and the presence of severe/moderately severe depression (defined as PHQ-9 ≥ 15) was replicated even after adjusting for various confounders, including age, gender, BMI, use of glucocorticoids, disease activity, and organ damage. These findings have important clinical implications, as they suggest that high NLR may serve as a useful marker to identify moderate and severe depression in SLE, regardless of disease activity. This finding could alert clinicians to screen SLE patients with high NLR for depression and refer them accordingly to mental health providers for further assessment and management. Further studies examining this association in SLE patients are necessary to confirm the findings of this study. This study is the first to evaluate the association between NLR and disease-specific HRQoL using the Lupus QoL. High NLR was associated with reduced scores in the domains of planning, physical health, and body image. These findings highlight the relationship of high NLR with HRQoL and indicate that NLR could serve as a useful tool for early recognition and prevention of poor quality of life in SLE. A strong association was also observed between high NLR and poor self-reported health. Specifically, a significantly higher percentage of patients in the high NLR group reported “poor” or “fair” health, with no patient reporting “excellent” self-rated health in this group. This highlights the value of NLR as a marker of the patients’ overall sense of well-being. Although the impact of disease activity as a potential cofounder cannot be excluded, these findings provide insights into the potential role of NLR as an indicator of the quality of life impairment, even when considering the influence of disease activity. Nonetheless, to better understand the relationship between NLR and quality of life measures in SLE, further research is warranted, adjusting for additional potential confounders such as disease activity.

Previous studies investigated the association between NLR and disease activity in SLE patients. These studies have suggested a variety of NLR cut-offs, as the optimal to indicate high disease activity, ranging from 2.065 to 2.94 [9–13]. This heterogeneity in values might reflect the differences in the characteristics of the population studied, the limited number of participants, and the difference in the definition of active disease in each study. The present study demonstrated that patients with NLR ≥ 2.73 had significantly higher scores of disease activity (SELENA-SLEDAI) and were more likely to have active disease (SELENA-SLEDAI ≥ 4). Han et al.[21], who also used the same NLR cut-off, found a significant association between high NLR and the presence of active disease, although a different definition of active disease was used (SLEDAI-2 K > 0), and the difference in the disease activity score, in general, was not significant. Furthermore, our study found that high NLR was associated with greater scores of PhGA, particularly those with a score of two or more, further supporting the relationship of NLR with disease activity.

The prevalence of high NLR in the population of our study (35%) was higher than in the general population (10%)[21], which is consistent with previous studies [8, 30]. This disparity in NLR values indicates that certain features of the disease affect NLR. In particular, NLR has been associated with key immunopathologic features of SLE: immune complex-mediated inflammation with the presence of anti-double-stranded DNA antibodies, type I interferon activity, circulating immunocomplexes, as well as neutrophil abnormalities, such as enrichment for low-density granulocytes and the neutrophil activation marker, calprotectin [21]. These associations support the relationship between high NLR and disease activity, underscoring the potential role of NLR as a disease activity marker.

The present study also provides evidence that high NLR is associated with organ damage as measured by the SLICC/ACR DI, suggesting that NLR may serve as a marker of disease-related damage. This finding is consistent with Abdulrahman et al.[31] who also demonstrated a positive correlation between SDI and NLR, among patients with lupus nephritis.

This study has limitations that should be acknowledged. Firstly, the relatively small number of patients included in the study may have limited the statistical power. Secondly, due to the cross-sectional design of this study, NLR values were measured only at a specific point in time and this may not reflect the longitudinal changes in NLR over time. Additionally, the study was conducted in Cyprus, a country with a predominantly Caucasian population; therefore, generalizing the results to patients from different racial groups should be done with caution. Moreover, it is important to note that there was no adjustment specifically for disease activity when assessing the poor quality of life, which does not allow us to fully exclude the potential confounding effect of disease activity on the observed association between NLR and quality of life. Finally, although the associations found were adjusted for glucocorticoid use, other factors such as the magnitude of the dose, other medications, and comorbidities may also influence the NLR and therefore act as potential confounding factors.

Further investigation with larger prospective cohort studies is needed to validate our findings and better characterize the relationship of NLR with HRQol, depression, and other outcomes, such as treatment response and mortality.

## Conclusion

In conclusion, the present study shows that in patients with SLE, high NLR is associated with severe depression, poorer self-rated health, impaired health-related quality of life, active disease, and the presence of underlying disease-related damage. Consequently, the results of this study can guide clinicians to recognize lupus exacerbations or active disease early and commence treatment accordingly. Regardless, clinical judgment and caution should be exercised in the interpretation of NLR, given that NLR has been shown to be increased in many inflammatory processes like sepsis [32], and it is advisable to be used with other indicators of disease activity. Furthermore, high NLR may signal the presence of underlying depression and impaired quality of life. These suggest its potential to serve as a valuable marker not only for disease activity but also to screen for depression and assess the quality of life. By recognizing these factors early, clinicians can provide more holistic care and improve patient outcomes. Nonetheless, these findings merit further research in larger cohorts.

## Data Availability

Data for this manuscript are not openly shared.
